# A patient with primary Burkitt’s lymphoma of the postnasal space: case report

**DOI:** 10.1186/1758-3284-4-33

**Published:** 2012-06-13

**Authors:** Tahwinder Upile, Waseem Jerjes, Jesuloba Abiola, Panagiotis Kafas, Ann Sandison, Zaid Hamdoon, Mohammed Al-Khawalde, Hani Radhi

**Affiliations:** 1 Department of Head and Neck Surgery, Chase Farm & Barnet NHS Trust, Enfield, UK; 2 Head & Neck Unit, University College London Hospital, London, UK; 3 Department of Surgery, School of Dentistry, Al-Yarmouk University College, Baghdad, Iraq; 4 Oral and Maxillofacial Surgery Unit, AL-Mustansirya University’s, Baghdad, Iraq; 5 UCL Department of Surgery, University College London, London, UK; 6 Leeds Institute of Molecular Medicine, Leeds, United Kingdom; 7 Department of Oral Surgery and Radiology, School of Dentistry, Aristotle University, Thessalonica, Greece; 8 Department of Pathology, Charring Cross Hospital, London, UK; 9 Oral and Maxillofacial Surgery Unit, Royal Medical Services, Amman, Jordan

## Abstract

**Introduction:**

Burkitt’s lymphoma is a highly aggressive lymphoma. The endemic form is present with Epstein - Barr virus. The most common sites are the mandible, facial bones, kidneys, gastrointestinal tract, ovaries, breast and extra-nodal sites. We present the first reported case of a primary Burkitt’s lymphoma of the postnasal space occurring in an elderly Caucasian male.

**Case presentation:**

A 72-year-old Caucasian male farmer presented with a 6-week history of a productive cough and a painless left sided cervical swelling. Examination of the neck revealed a 5 cm by 5 cm hard mass in the left anterior triangle. A CT scan of the head and neck showed a soft tissue swelling in the postnasal space. Histology of the postnasal space mass showed squamous mucosa infiltrated by a high grade lymphoma.

Immunohistochemical staining and *in situ* hybridisation confirmed the tumour to be Epstein - Barr virus Ribonucleic acid negative suggesting this was a rare sporadic form of the tumour presenting in a location that is atypical for the clinical subtype and age of the patient.

**Conclusion:**

This is the first reported case of sporadic Burkitt’s lymphoma of the postnasal space of an elderly Caucasian male in the absence of Epstein - Barr virus or human immunodeficiency virus infection and further serves to illustrate the diversity of histological subtypes of malignancies that may develop at this concealed site.

## Introduction

Burkitt’s lymphoma is a highly aggressive lymphoma that appears to occur in three histologically and phenotypically identical but clinically distinct forms; endemic, sporadic and immunodeficiency-associated. The endemic form is present with Epstein - Barr virus (EBV) in nearly all cases. It is most commonly found in children with a male preponderance [1].

The most common sites are the mandible, facial bones, kidneys, gastrointestinal tract, ovaries, breast and extra-nodal sites. Sporadic Burkitt’s is found worldwide with the abdomen being most commonly involved. EBV is involved in up to 30% of cases [1-3].

Cases have also been reported in which a Burkitt’s-like lymphoma is associated with HIV [2-4], congenital immunodeficiency as well as post transplant patients [5-8].

We present the first reported case of a primary Burkitt’s lymphoma of the postnasal space occurring in an elderly Caucasian male.

## Case presentation

All treatments were given according to standard accepted guidelines in accordance with the hospital R&D and ethical review committees, patient consent was obtained prior to publication. A 72-year-old Caucasian male farmer presented with a 6-week history of productive cough and painless left sided cervical swelling. He had given up smoking cigarettes fifteen years previously. Examination of the neck revealed a 5 cm by 5 cm hard mass in the left anterior triangle by the angle of the mandible with no other palpable masses.

Fibre-optic naso-endoscopy revealed a 3 cm by 3 cm mass extending from his left Eustachian cushion towards the midline. History and examination were otherwise unremarkable.

**Figure 1:**
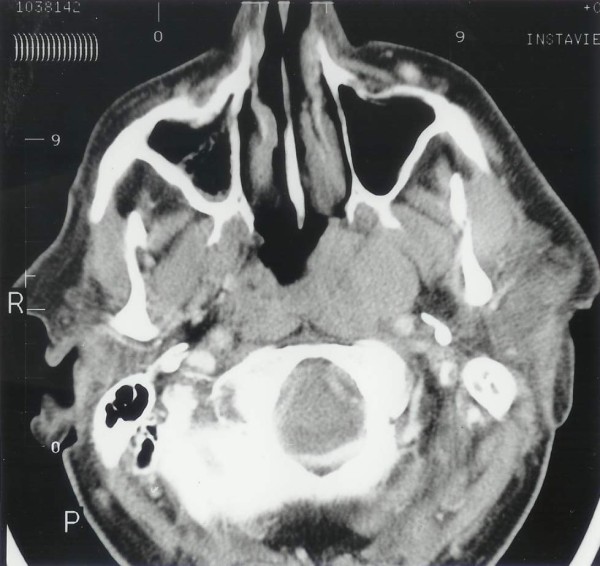
Computed tomography scan showing a well circumscribed uniform density mass on the left side of the posterior wall of the nasopharynx.

**Figure 2:**
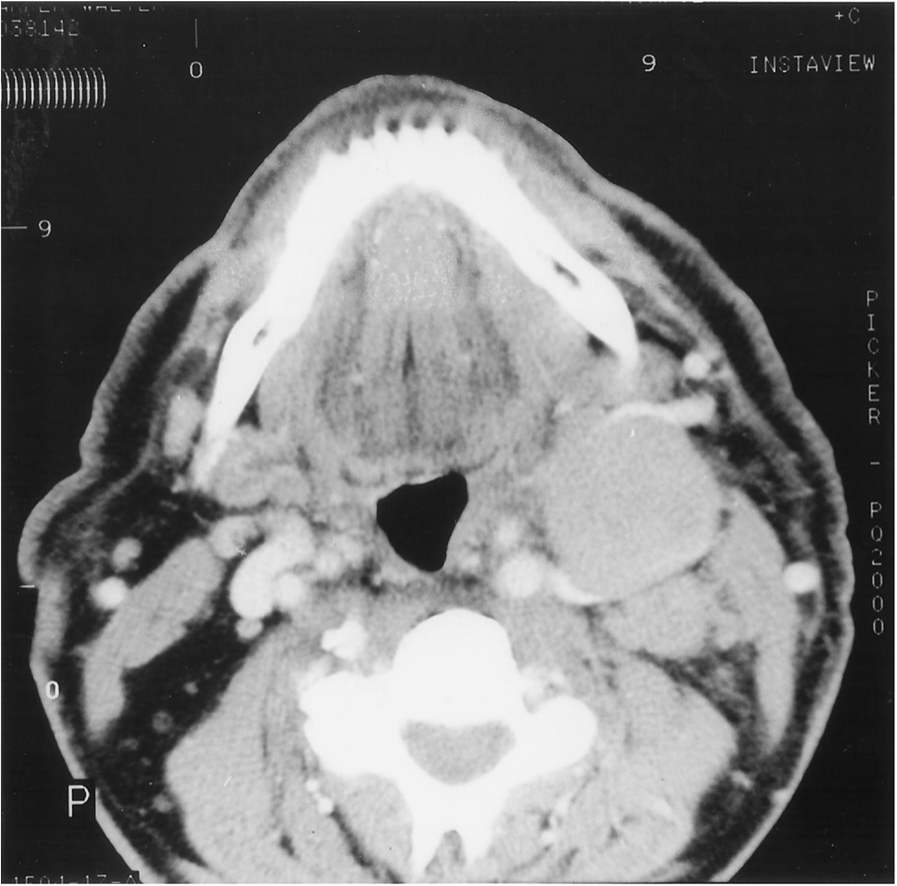
Computed tomography scan showing a well circumscribed lymph node in the left side of the neck.

A CT scan of the head and neck showed a soft tissue swelling in the postnasal space, with extensive lymphadenopathy along the carotid sheath and deep to the sternomastoid (Figure 1). The lowest enlarged lymph node being at the level of the hyoid bone. The largest lymph node measured 3.5 cm in diameter (Figure 2). CT of the thorax, abdomen and pelvis showed no other abnormality.

Endoscopically directed biopsies of the postnasal space mass and a fine needle aspirate (FNA) of the neck lump were performed under general anaesthesia. And on microscopy showed atypical lymphoid cells on a background of foamy macrophages and cellular debris.

**Figure 3:**
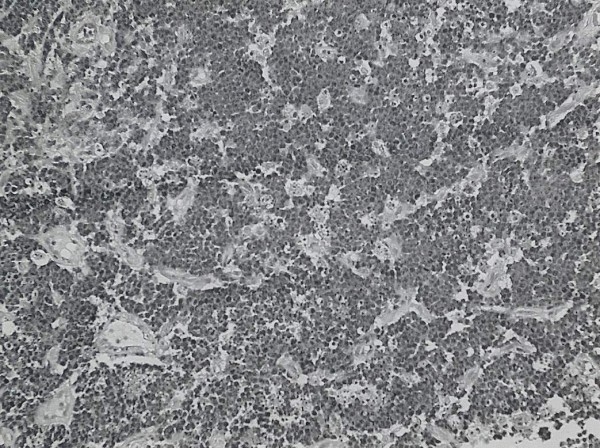
**Histopathology section showing squamous mucosa infiltrated by a high-grade lymphoma (H&E,** ***x*** **2 objective).**

**Figure 4:**
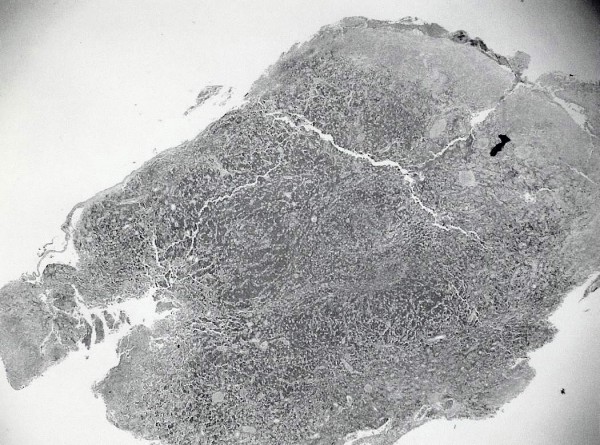
Histopathology section, the infiltrate is composed of a monomorphic population of medium sized cells showing a typical starry sky pattern (H&E, low objective).

**Figure 5:**
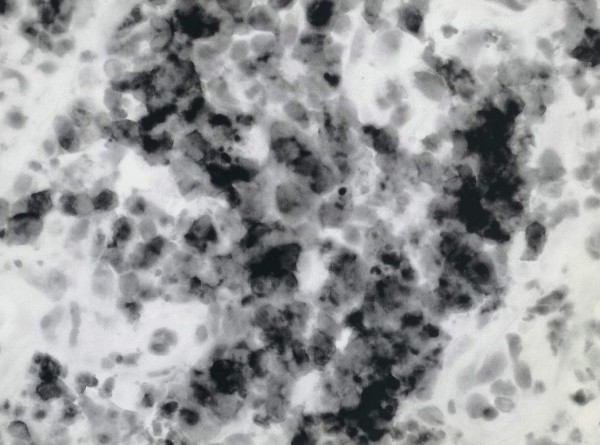
Histopathology section, the lymphoid cells show a “squared off” appearance along with numerous mitotic figures and macrophages (H&E, x40 objective).

Histology of the postnasal space mass showed squamous mucosa infiltrated by a high grade lymphoma comprising a mono-morphic population of tightly packed, medium sized lymphoid cells exhibiting a very high mitotic rate along with numerous characteristic “starry sky” macrophages (Figures 3, 4 and 5). The immunohistochemical profile of the tumour was in keeping with a Burkitt’s lymphoma. A diagnosis of Burkitt’s lymphoma with involvement of the cervical lymph nodes was made.

Immunohistochemical staining and *in situ* hybridisation for Epstein - Barr virus (EBV) revealed the tumour to be EBV Ribonucleic acid (RNA) negative, EBNA2 (monoclonal) negative and LMP1 (monoclonal) negative, confirming there was no previous infection with the Epstein Barr Virus. Human immunodeficiency virus (HIV) serology was negative confirming that the lymphoma was not HIV-associated.

The patient was treated with a CODOX-M chemotherapy regime (Cyclophosphamide 350 mg Day 2–5, Cytarabine 70 mg IT DI, 3, Methotrxate 3 g/m2 DIO with folinic acid rescue) and remains in remission after five years.

## Discussion

Both sporadic and endemic forms of Burkitt’s lymphoma present primarily in childhood [8]. An abdominal tumour is the single most common presentation in sporadic Burkitt’s lymphoma while a jaw mass is the usual mode of presentation in the endemic form. Review of the literature suggests nasopharyngeal Burkitt’s lymphoma occurs only in childhood [9-11].

Preferred sites for sporadic Burkitt’s include the bowel, the ovaries, the mesentery and retroperitoneal lymph nodes [5]. A significant number, however, do present in the nasopharynx and Waldeyer’s ring [12]. In one published series just over one half of all childhood lymphoblastic and non-endemic Burkitt’s lymphomas were extra-nodal at presentation with one third occurring in the aero-digestive tract [13].

It is not clear why this particular patient had sporadic nasopharyngeal Burkitt’s lymphoma in this highly atypical site. To our knowledge, this is the first reported case in the world literature of Burkitt’s lymphoma in an elderly Caucasian male.

## Conclusions

This case is unusual as it is a sporadic tumour in an elderly presenting at a site favoured by endemic paediatric tumours.

In addition, this is the first reported case of sporadic Burkitt’s lymphoma of the postnasal space of an elderly Caucasian male in the absence of EBV or HIV infection and further serves to illustrate the diversity of histological subtypes of malignancies that may develop at this concealed site.

## Patient consent

Written informed consent was obtained from the patient for publication of this case report and accompanying images. A copy of the written consent is available for review by the Editor-in-Chief of this journal.

## Competing interest

The authors declare that they have no competing interests.

## Authors’ contributions

All authors contributed to conception and design, carried out the literature research, manuscript preparation and manuscript review. All authors have read and approved the final version of the manuscript.
